# Fossils of an endangered, endemic, giant dipterocarp species open a historical portal into Borneo's vanishing rainforests

**DOI:** 10.1002/ajb2.70036

**Published:** 2025-05-08

**Authors:** Teng‐Xiang Wang, Peter Wilf, Antonino Briguglio, László Kocsis, Michael P. Donovan, Xiaoyu Zou, J. W. Ferry Slik

**Affiliations:** ^1^ Department of Geosciences and Earth and Environmental Systems Institute Pennsylvania State University University Park 16802 Pennsylvania USA; ^2^ IUCN/SSC Global Tree Specialist Group Botanic Gardens Conservation International Richmond TW9 3BW UK; ^3^ Dipartimento di Scienze della Terra, dell'Ambiente e della Vita Università degli Studi di Genova Genoa 16132 Italy; ^4^ Institute of Earth Surface Dynamics, Faculty of Geosciences and Environment University of Lausanne Lausanne 1015 Switzerland; ^5^ Geological Collections, Gantz Family Collections Center Field Museum of Natural History Chicago 60605 Illinois USA; ^6^ Institute of Geophysics and Planetary Physics, Scripps Institution of Oceanography University of California San Diego La Jolla 92093 California USA; ^7^ Faculty of Science Universiti Brunei Darussalam Gadong BE1410 Brunei Darussalam

**Keywords:** Asian rainforests, Borneo, cuticles, Dipterocarpaceae, endangered species, paleobotany, peatlands

## Abstract

**Premise:**

Asia's wet tropical forests face a severe biodiversity crisis, but few fossils record their evolutionary history. We recently discovered in situ cuticles on fossil leaves, attributed to the giant rainforest tree *Dryobalanops* of the iconic Dipterocarpaceae family, from the Plio‐Pleistocene of Brunei Darussalam (northern Borneo). Studying these specimens allowed us to validate the generic identification and delineate affinities to living dipterocarp species.

**Methods:**

We compared the leaf cuticles and architecture of these fossil leaves with the seven living *Dryobalanops* species.

**Results:**

The cuticular features shared between the fossils and extant *Dryobalanops*, including the presence of giant stomata on veins, confirm their generic placement. The leaf characters are identical to those of *D. rappa*, an IUCN red‐listed Endangered, northern Borneo endemic. The *D. rappa* monodominance at the fossil site, along with *Dipterocarpus* spp. leaf fossils, indicates a dipterocarp‐dominated forest near the mangrove‐swamp depocenter, most likely in an adjacent peatland.

**Conclusions:**

The *Dryobalanops rappa* fossils are the first fossil evidence of a living endangered tropical tree species and show how analysis of in situ cuticles can help illuminate the poorly known floristic history of the Asian tropics. This discovery highlights new potential for fossils to inform heritage values and paleoconservation in Southeast Asia.

The Asian tropics harbor extraordinary plant diversity comparable to the Neotropics (Slik et al., 2015; Raven et al., 2020). The large island of Borneo lies within the Sundaland biodiversity hotspot and has exceptional plant diversity and endemism (Myers et al., 2000; Neo et al., 2021). Lowland Borneo, in particular, is recognized as the ecoregion with the highest estimated number of vascular plant species worldwide (~10,000; Kier et al., 2005). Nevertheless, like much of the Asian wet tropics, Borneo faces severe extinction risks from anthropogenic pressures (Sodhi et al., 2004; Malhi et al., 2014). Vast areas have been cleared for logging or converted for agriculture, resulting in ~30% forest loss from the 1970s to 2010 (Curran et al., 2004; Gaveau et al., 2014). Protecting Borneo's endangered species and their habitats against extinction is a widely acknowledged conservation priority (Myers et al., 2000), but historical data from fossils are very rare.

Plant fossils provide fundamental knowledge about the evolutionary history and paleoconservation of forested biomes (Kooyman et al., 2014, 2020; Carvalho et al., 2021; Wilf and Kooyman, 2025). However, paleobotanical (referring to macrofossils) research throughout the Asian wet tropics remains limited and largely insufficient to understand the assembly of its threatened living forests (Kooyman et al., 2019; Wilf et al., 2022; Spagnuolo et al., 2024), although a great deal is known from palynological data (Morley, 2024). This situation contrasts with Australia, the Neotropics, and the African tropics, where substantial paleobotanical discoveries over the past few decades have vastly improved historical understanding of the living rainforests (Hill, 1994; Carvalho et al., 2021; Jaramillo, 2023; Pan et al., 2023; Slodownik, 2024). The Gondwana Rainforests of Australia and The Wet Tropics of Queensland World Heritage sites serve as leading examples of how paleobotanical evidence contributes directly to the recognition of World Heritage values, promoting conservation of ancient, now threatened plant lineages and entire ecosystems (UNESCO, 2001, 2023; Wilf and Kooyman, 2025). There are no comparable conservation areas in the Asian wet tropics.

The dipterocarp family (Dipterocarpaceae) comprises >500 tree species and structurally defines the lowland rainforests of the Asian tropics (Ashton, 1982; Corlett, 2007; Ashton et al., 2021). Borneo is the center of dipterocarp diversity and endemism, with 162 of its 269 species endemic (Bartholomew et al., 2021). Dipterocarp trees, most of them extremely tall, hold primary ecological importance due to their roles in forest stratification, mast fruiting, and ectomycorrhizal associations, in addition to the economic significance of their timber and resins and aesthetic value (Ashton et al., 2021). About 357 (67%) of the world's dipterocarp species are threatened with extinction, including 99 Bornean endemic species (Bartholomew et al., 2021; Khoo et al., 2023). Nevertheless, definitive macrofossil evidence of dipterocarps from Borneo and the broader Asian wet tropics is scant (Wilf et al., 2022). Many ambiguous leaf‐ and wood‐fossil species were established on the basis of insufficient morphological characters (Ashton et al., 2021), and their connections with living species are unresolved.

The dipterocarp genus *Dryobalanops* (kapur) contains seven canopy‐emergent (≤80 m height) tree species that are endemic to Borneo, Sumatra, and the Malay Peninsula (Ashton, 1982, 2004; Figure 1), all assessed as threatened or in decline (Bartholomew et al., 2021; IUCN, 2024). However, the *Dryobalanops* fossil record is scarce and ambiguous, represented by Eocene pollen from India and macrofossils entirely of fossil wood (Biswas et al., 2019; Bansal et al., 2022; Figure 1; Appendix S1). Definitive fossil records of this genus, potentially linked to its extant species, are necessary to better understand its evolutionary history.

**Figure 1:**
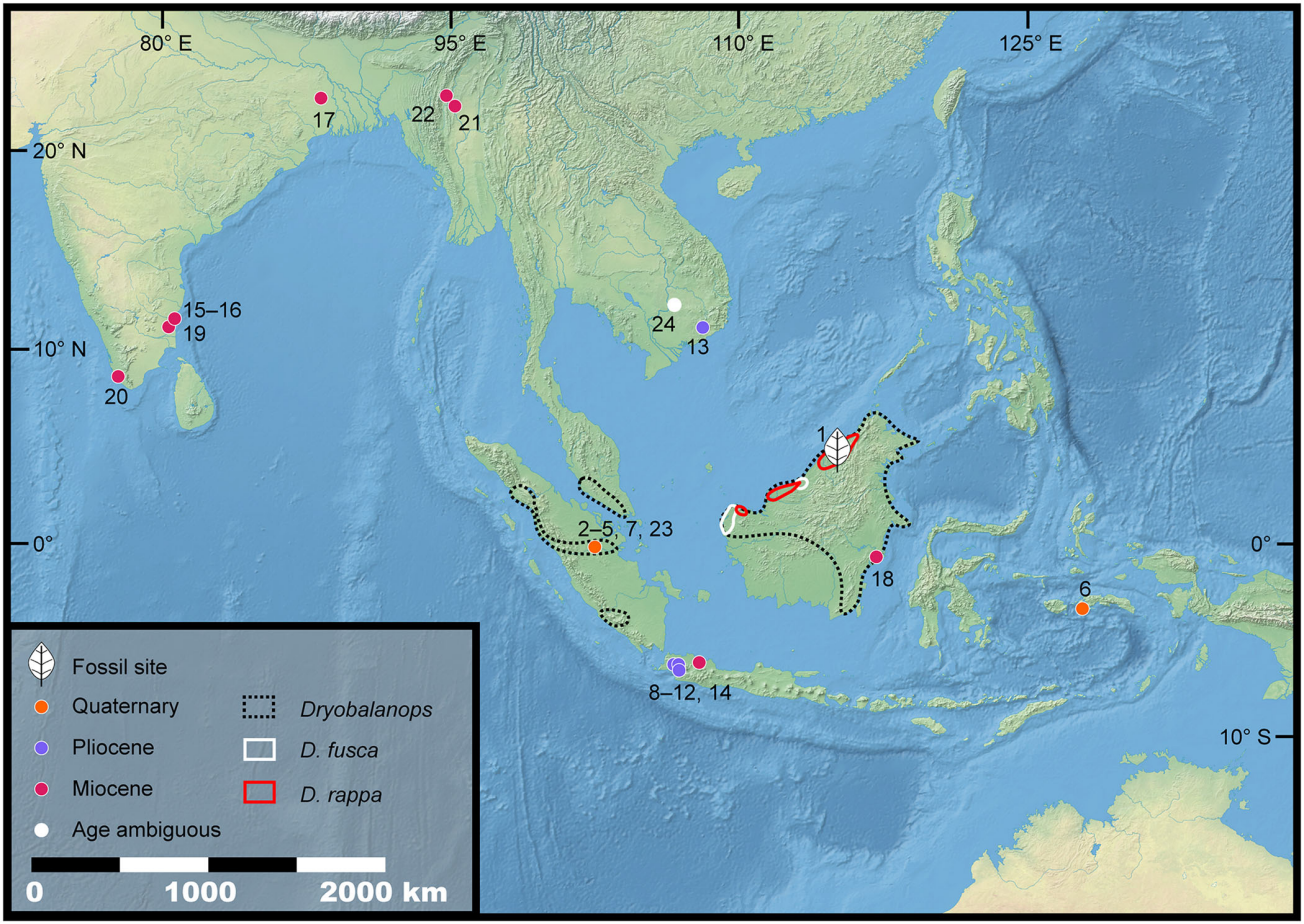
Macrofossil wood records (*Dryobalanoxylon* spp.), the *Dryobalanops* leaf fossils in this study (leaf symbol), and the extant distribution of *Dryobalanops*. Scale corresponds to distances at the equator. Numbers correspond to Appendix S1. Base map is from Natural Earth II, https://www.naturalearthdata.com. Data sources are published literature for the fossils (Appendix S1) and GBIF for extant range data (urls: https://doi.org/10.15468/dl.rcvpj7; https://doi.org/10.15468/dl.6duuxq; https://doi.org/10.15468/dl.nrm67g).

In their recent paper on the first fossil floras from Brunei Darussalam, a sultanate on the northern coast of Borneo, Wilf et al. (2022) recognized abundant (three‐quarters of all identifiable specimens) leaf compressions of *Dryobalanops* (Figures 1 and 2) at a Plio‐Pleistocene fossil site in the village of Kampong Lugu. Sedimentological, paleobotanical, and palynological data indicated that the leaves were deposited in a mangrove swamp after transportation from an adjacent, dipterocarp‐dominated coastal rainforest (Wilf et al., 2022). The fossils were identified based on leaf architecture alone, requiring their distinction from a long list of speciose genera whose leaves have similar, densely spaced secondary venation (i.e., taxa in Myrtaceae, Sapotaceae, Ochnaceae, Moraceae, Vochysiaceae, and Calophyllaceae), and were suggested to be similar to the living species *D. fusca* (Wilf et al., 2022).

**Figure 2:**
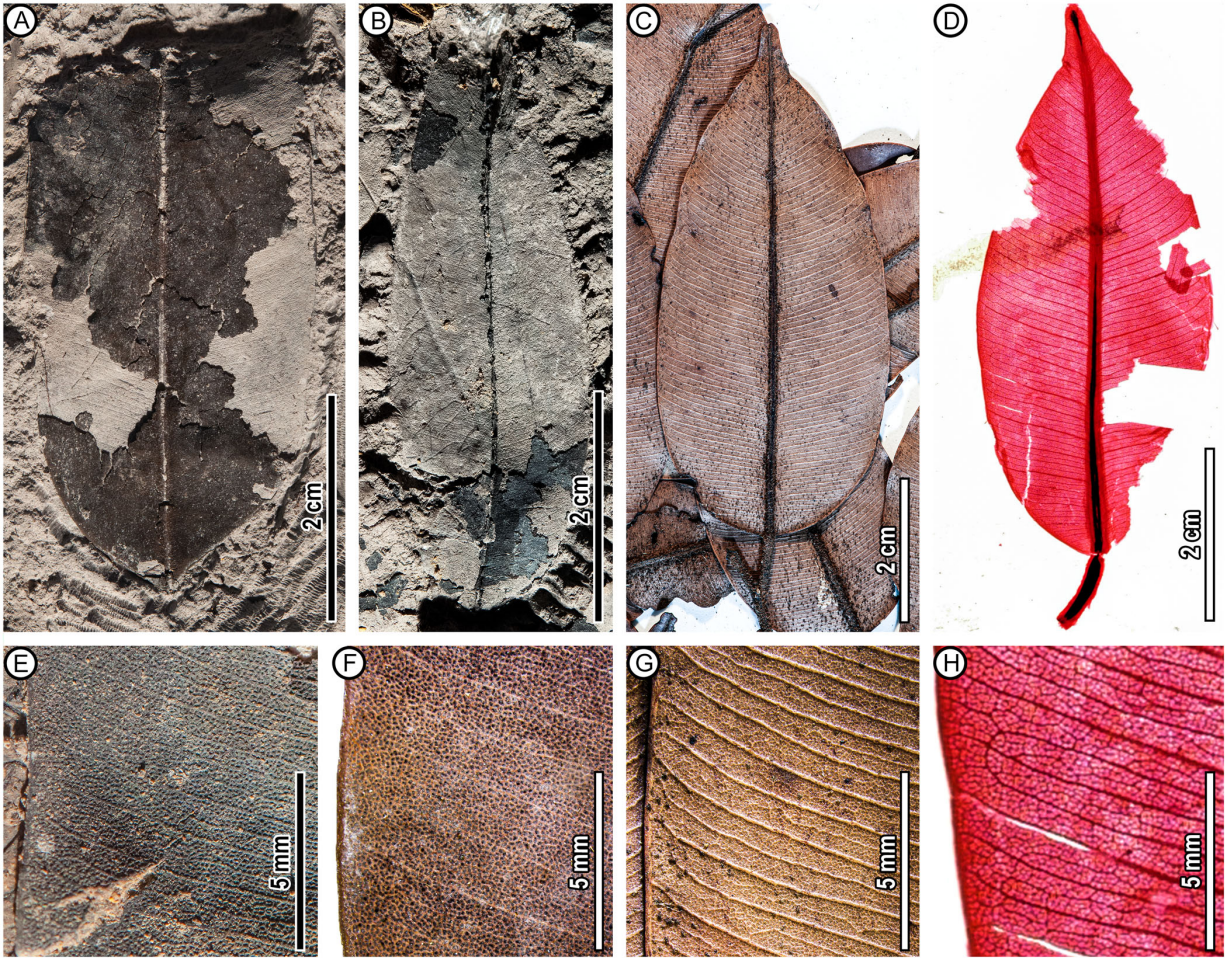
Leaf architecture of fossil (A, B, E) and comparable extant (C, D, F–H) *Dryobalanops* species. (A) UBDH F00266, a source of cuticle (see Figures 4 and 5). (B) UBDH F00332. (C) *D. rappa* leaf abaxial surface (S. Tong S. 34166, 1974, Sarawak, A02566817). (D) Cleared leaf of *D. fusca* (Jack A. Wolfe USGS National Cleared Leaf Collection, Wolfe 7923. Source voucher: A, Ariffin 9625, Sarawak, downloaded from the image dataset of Wilf et al., 2021). (E) UBDH F00192a, showing rows of polygonal areolae and intramarginal vein close to the margin, as seen in some extant species (F–H). (F, G) *D. rappa* leaf adaxial (F; Source: North Borneo Forestry Department 2467, 1932, Sabah, A02566809) and abaxial (G; enlargement of panel C at left margin) surfaces, showing dense and parallel secondary and well‐developed intersecondary veins, rows of polygonal areolae (darkened in F) and intramarginal vein close to the margin, all features comparable to the fossils (E). (H) Enlargement of panel D.

In light of the well‐known ecological and conservation significance of dipterocarps, their limited fossil record, and the general issue of convergence in leaf architecture, corroborative evidence is needed to confirm that these important leaf fossils were correctly identified to genus and to refine their affinities to living species. We recently discovered in situ cuticles on some specimens, providing numerous new characters that allow us here to evaluate the generic assignment and make comprehensive comparisons with all extant *Dryobalanops* species. The combined data from leaf architecture and cuticular morphology confirm the fossils' botanical affinity to *Dryobalanops*, and we assign them to the living endangered species *D. rappa*, which forms dominant stands today in regional peatlands of Brunei and Malaysian Borneo (Ashton, 2004; Hamidi et al., 2019). Our discovery represents the first unequivocal fossil record of *Dryobalanops* leaves and the first macrofossil evidence worldwide of any living, endangered tropical tree species, unlocking new opportunities for paleobotanical understanding and paleoconservation of living tropical rainforests.

## MATERIALS AND METHODS

The fossil leaves studied here were reported by Wilf et al. (2022) as *Dryobalanops* sp. BR03 from Kampong Lugu, Tutong District, Brunei Darussalam (N 4.87582°, E 114.80229°). The fossiliferous strata belong to an unnamed lithologic unit that horizontally overlies the marine, late Miocene Miri Formation with a ~30° angular unconformity, indicating a substantially younger age for the fossils. This rock unit was discovered and described by Wilf et al. (2022) and has not yet been integrated into the local geological framework (e.g., Kocsis et al., 2022). Palynological data bracketed a potential age range of early Miocene to Pleistocene (Wilf et al., 2022), and the authors considered the age of the Kampong Lugu fossil flora as most likely Plio‐Pleistocene, which we maintain here. Additional taxa included two species of *Dipterocarpus* and possible *Shorea* (Dipterocarpaceae), as well as Melastomataceae. Wilf et al. (2022) also reported a single leaf of *Dryobalanops* sp. BR03 (without preserved cuticle) from the late Miocene‐Pliocene Berakas Beach locality, associated with a winged *Shorea* fruit and leaves of *Dipterocarpus*, Melastomataceae, *Ziziphus*, *Rhaphidophora*, cf. Myrtaceae, and cf. Malvaceae. This occurrence suggests a pre‐Pleistocene history of the species studied here.

Fossil cuticle preparation followed conventional methods (Dilcher, 1974). Small pieces (roughly 0.5 cm × 0.5 cm) of coalified fossil‐leaf compressions were picked and treated with 30% HCl for 2 d. The use of HF was not necessary, given minimal sediment adhesion. For maceration, we used 30% H_2_O_2_ at room temperature for 24 h. The cuticle pieces became yellowish, transparent, and easily separable, indicating the completion of the maceration. Household bleach is a convenient alternative medium for maceration, requiring only minutes per treatment, but it tends to make the thin abaxial cuticle extremely curly, fragile, and difficult to mount on a slide. The cuticles were then washed with distilled water. They were separated into abaxial and adaxial components and cleaned with dissecting needles under a Nikon SMZ1500 stereomicroscope (Nikon, Melville, New York, USA) to remove the tissue remnants on the inner surfaces of both cuticles, after which the cleaned cuticles were ready for mounting.

To obtain cuticles from extant *Dryobalanops* leaves, we requested samples from Naturalis Biodiversity Center, Leiden (L), the Netherlands of small leaf fragments (~1 cm^2^) from all seven species, avoiding the margin and midvein. Cuticle preparation followed Huang et al. (2018). The leaf fragments were treated with glacial acetic acid plus 30% H_2_O_2_ in a 60°C water bath. This process softened and dissolved the mesophyll and required 8–30 h, depending on the species. The treatment was stopped when the leaf fragments became transparent, the cuticles tended to peel off, and the mesophyll was easily removed using hand tools. The samples were washed several times with distilled water and then transferred to a stereomicroscope. Dissecting needles were used to separate the abaxial and adaxial cuticles, and the remaining mesophyll was scraped off gently, providing clean cuticles that were ready to mount.

To prepare slides for transmitted light microscopy (modern and fossil specimens), cleaned cuticles were stained with 5% Safranin O, dehydrated with glycerin, and mounted on slides with glycerin jelly. To prepare slides for epifluorescence microscopy (fossil specimens), the cuticles were dehydrated with graded baths of ethanol and then mounted on slides with Cytoseal XYL (Epredia, Kalamazoo, Michigan, USA). The slides were observed using an X‐Cite 120 epifluorescence illumination unit (EXFO Electro‐Optical Engineering, Quebec City, Quebec, Canada) with a long‐pass green filter on a Nikon LV100 Eclipse microscope and photographed with a DS‐Ri1 camera and Nikon NIS Elements Basic version 3.0 at the Pennsylvania State University (PSU) Paleobotany Laboratory, Pennsylvania, USA. We used Adobe Photoshop version 23.3.1, from the Adobe Creative Cloud Suite version 6.1.0.587.7 to generate stacked images. Stomatal and epidermal characteristics were measured using ImageJ (Schneider et al., 2012; https://imagej.net/ij/) on light microscopic images (Table 1; Appendix S2). For Scanning Electron Microscopy (SEM) sample preparation, cleaned cuticles were dehydrated with a graded series of ethanol baths and mounted on SEM stubs as they air dried. The samples were processed using a Bal‐Tec SCD‐050 Sputter Coater (Bal‐Tec, Los Angeles, California, USA) and observed under a Zeiss Sigma VP‐FESEM microscope (Zeiss, Dublin, California) at the PSU Huck Institutes of the Life Sciences Microscopy Facility. All fossil materials are deposited at the Herbarium of Universiti Brunei Darussalam (UBDH) and were studied while on loan to PSU. We followed Dilcher (1974) and Barclay et al. (2007) for cuticle terminology.

**Table 1:** Comparison of *Dryobalanops* fossil and extant species.

*Dryobalanops* species	Leaf morphology		Normal stomata (10 measurements)				Giant stomata (two measurements)			Abaxial trichome base density (*N*/mm^2^)
	Leaf shape	Leaf length and width (cm)	Pore length (μm)	Guard cell length (μm)	Guard cell width (μm)	Density (*N*/mm^2^)	Pore length (μm)	Guard cell length (μm)	Guard cell width (μm)	
**Fossils, this study**	**Elliptic or ovate‐lanceolate**	**4.0–7.7 × 0.7–5.0**	**6–8 (7)**	**16–22 (18)**	**5–6 (5)**	**841**	**14–18 (16)**	**32–42 (37)**	**8**	**76**
***D. rappa***	**Ovate‐lanceolate**	**6–11 × 2.5–4**	**6–8 (7)**	**14–23 (16)**	**5–8 (6)**	**678**	**11–14 (12)**	**30–33 (31)**	**9–11 (10)**	**30**
*D. aromatica*	Broadly ovate	4–6 × 2–4	7–10 (9)	15–18 (17)	4–7 (5)	583	15–19 (17)	38–42 (40)	10–10 (10)	36
*D. beccarii*	Ovate‐lanceolate to oblong‐lanceolate	5–8 × 1–3	5–9 (7)	11–17 (14)	4–7 (5)	587	14–18 (16)	37–40 (38)	11	7
*D. fusca*	Broadly lanceolate	5–10 × 2–4	7–14 (12)	20–31 (27)	8–11 (10)	254	19–21 (20)	42–43 (43)	13–15 (14)	95
*D. keithii*	Narrowly oblong, lanceolate or oblanceolate	14–33 × 5–10	6–9 (7)	13–19 (16)	5–9 (7)	498	10–12 (11)	24–25 (25)	8	21
*D. lanceolata*	Narrowly lanceolate	7–10 × 2–3.5	7–11 (9)	18–22 (20)	6–8 (7)	459	16–22 (19)	38–42 (40)	12–16 (14)	83
*D. oblongifolia*	Oblong	6–20 × 4.5–5(–6.5)	5–8 (6)	15–19 (17)	6–8 (7)	425	15–17 (16)	33–35 (34)	10–11 (10)	4

*Notes*: Leaf morphology data are from Ashton (2004) and Wilf et al. (2022). Measurements of stomatal complexes and trichome bases, derived from this study, are rounded to integer values. Values in parentheses are medians.

Herbarium specimens of *Dryobalanops rappa* and *D. fusca* were obtained on loan from the Harvard University Herbaria, Massachusetts, USA, for detailed photography at PSU. A Nikon D90 camera with a 60 mm macro lens was used to capture the image in Figure 2C. Images for Figure 2F and G were obtained using a stereomicroscope. Epifluorescence microscopy was employed for Appendix S3. Fossil cuticles were also found on other, unidentified leaf fossils from the same site (Appendix S4), showing potential for further study of this fossil flora.

## RESULTS

### Systematics

**Family**―Dipterocarpaceae Blume

**Genus**―*Dryobalanops* C.F.Gaertn.

**Species**―*Dryobalanops rappa* Becc.

Former treatment: *Dryobalanops* sp. BR03 (Wilf et al., 2022: 23).

**Specimens reexamined**―UBDH F00266 (Figures 2A, 4A–I, and 5), UBDH F00286 (Figure 4J–L), UBDH F00192 (Figure 2E), UBDH F00332 (Figure 2B).

**Locality and age**―Kampong Lugu, Tutong District, Brunei Darussalam (N 4.87582°, E 114.80229°), Plio‐Pleistocene.

**Repository**―Herbarium of Universiti Brunei Darussalam (UBDH).

**Description**―*Blade* elliptic to ovate‐lanceolate, folded longitudinally; size microphyll (to 7.7 cm length). Apex broad‐acuminate; base straight or cuneate. Major secondary veins numerous and closely spaced; intersecondaries alternating with secondaries; secondaries and intersecondaries straight, joining intramarginal veins that run very close to the margin (Figure 2A, B). Tertiary veins regular reticulate, forming two to five rows of well‐defined rectangular to polygonal fields packed between each secondary‐intersecondary vein pair (Figure 2E). See Wilf et al. (2022) for further descriptions and illustrations of the fossils' leaf architecture.

**Figure 3:**
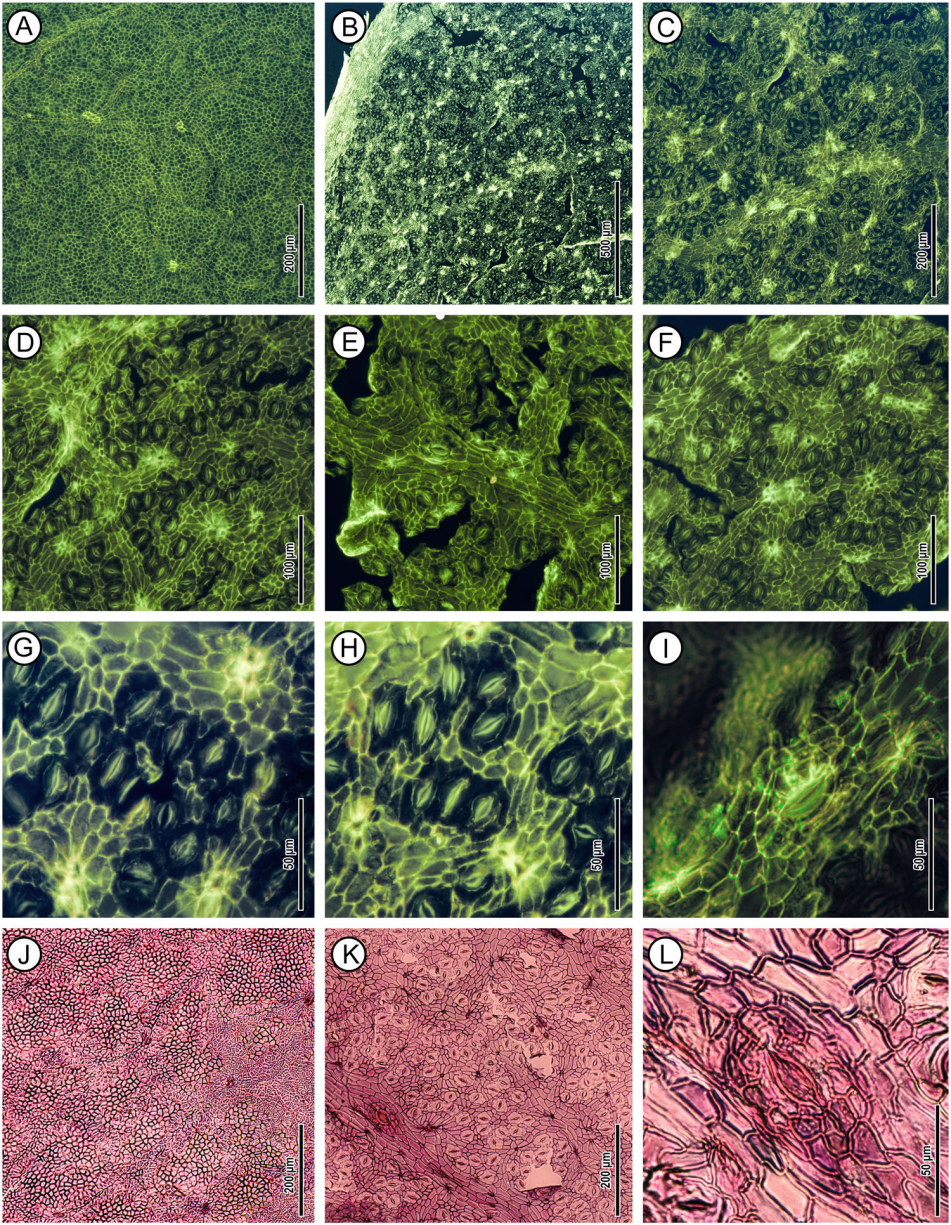
Fossil *Dryobalanops rappa* cuticles under epifluorescence (A–I, UBDH F00266) and transmitted light (J–L, UBDH F00286). (A, J) Adaxial cuticle in wide view. (B–I, K, L) Abaxial. (B, C) Wide view showing stomata densely packed between parallel secondary‐intersecondary veins. (D–F, K) Detail, scattered groups of regular stomata, giant stomata, trichome bases, and portions of secondary/intersecondary and tertiary veins. (G, H) Detail, regular stomata packed into small areolae. (I, L) Giant stoma, with numerous subsidiary cells.

**Figure 5:**
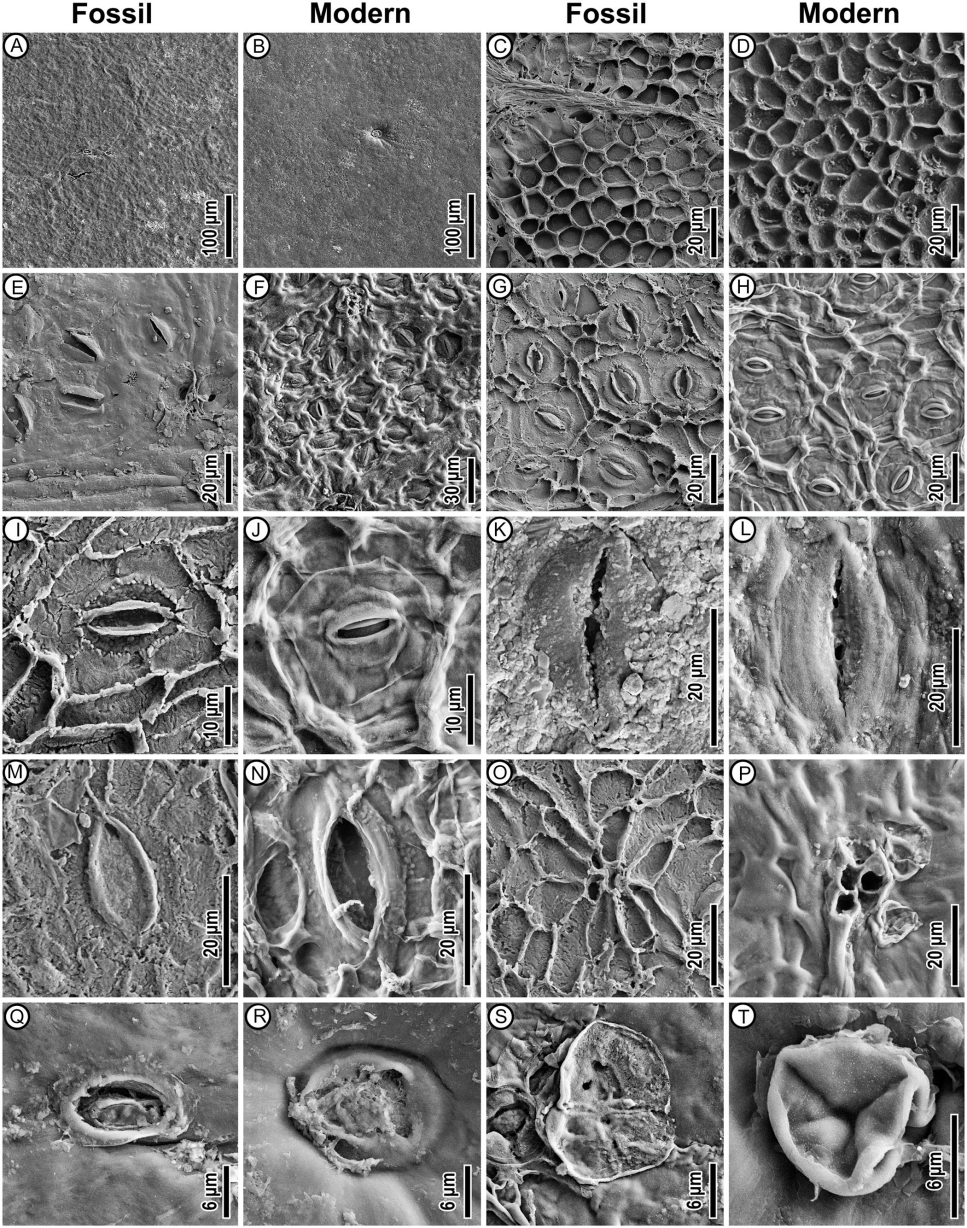
Fossil and extant *Dryobalanops rappa* cuticles under the scanning electron microscope. First and third columns are a fossil specimen (UBDH F00266); second and fourth columns are an herbarium specimen (Brunei, L.2430702). (A, B) Outer adaxial cuticle, showing nearly glabrous surface and a single‐celled trichome base. (C, D) Inner adaxial cuticle, showing faviform pattern. (E, F) Outer side of abaxial cuticle, showing regular stomata and trichome bases. (G, H) Inner side of abaxial cuticle, showing a cluster of regular stomata. (I, J) Regular stoma, inner abaxial cuticle. (K, L) Giant stoma, outer abaxial cuticle. (M, N) Giant stoma, inner abaxial cuticle. (O, P) Three‐celled trichome base on abaxial cuticle (O, inner side; P, outer side). (Q, R) Rounded trichome bases, outer adaxial cuticle. (S, T) Peltate hair, outer side of abaxial cuticle.

*Cuticle* hypostomatic. *Adaxial cuticle* moderately thick. Non‐venous epidermal cell shape isodiametric and variously tetragonal, pentagonal, or hexagonal; size inconsistent; anticlinal cell wall outline rounded, with no undulation (Figure 3A, J). Venous epidermal cells slightly elongated, generally similar to non‐venous epidermal cells, connecting to form areolae (Figure 3A). Cuticle inner surface pattern faviform, without special ornamentation (Figure 5C). Trichome bases rare, shape rounded, rim visible on the cuticle's outer surface (Figure 5Q).

*Abaxial cuticle* moderately thin. Venous epidermal cells elongated or rectangular, linearly arranged, connecting to form areolae encircling numerous stomata between adjacent secondary or intersecondary veins (Figure 3B–D); the areolae correspond to the macroscopic polygonal fields (Figure 2E). Anticlinal cell wall outlines rounded, with no undulation (Figures 3G,H and 5C). Non‐venous epidermal cells surrounded by stomata; shape isodiametric and variously polygonal (Figure 3G, H). Cuticle inner surface without special ornamentation. Trichome bases common; density ~76/mm^2^; shape rounded, appearing single‐celled, or in multiple‐celled clusters on the rims of the areolae (Figure 5O), close to the venous epidermal cells of major and fine veins; trichome bases much brighter than other structures under epifluorescence (Figure 3D–F). Peltate hair remnants present (Figure 5S). Stomatal complexes confined to areolae (Figure 3G, H), shape oval or narrowly oval; pore length 6–8 μm, median 7 μm; guard cell length 16–22 μm, median 18 μm; guard cell width 5–6 μm, median 5 μm; stomatal density ~841/mm^2^ (Table 1); type cyclocytic (Figure 5I); subsidiary cells generally <10 but difficult to count because they are weakly segregated, less fluorescent, and stained poorly (Figure 3G, H). Apertures preserved open. Special surface ornamentation not observed on guard cells. Giant stomatal complexes present only on primary, secondary, and intersecondary veins, shape narrowly oval, cyclocytic with guard cells and more than ten subsidiary cells (Figures 3I, L and 5K–N); pore length 14–18 μm; guard cell length 32–42 μm; guard cell width ~8 μm; special surface ornamentation not observed. Domatia, cork‐warts, and glands not observed.

### Cuticular features of extant *Dryobalanops* leaves

The cuticular structures of Dipterocarpaceae are known only from a few publications that show cuticle images of *Shorea* and *Dipterocarpus* (Khan et al., 2015; Chen et al., 2021). Little is known about the leaf epidermal features of *Dryobalanops*, other than the presence of hairs on certain species (Ashton, 2004). The present study is the first to investigate the shared cuticular features of *Dryobalanops* and their taxonomic significance.

All seven *Dryobalanops* species have hypostomatic leaves. The adaxial epidermal cells have thickened anticlinal walls and variable polygonal shapes, which become slightly elongated over the veinlets (Figure 4). In some species, the veinlets form well‐defined areolae between secondary‐intersecondary veins (Figure 4F, O). Adaxial trichome bases are rare. The abaxial cuticles show numerous stomata in clusters enclosed by rows of areoles between secondary‐intersecondary veins, matching the densely packed polygonal fields observed macroscopically (Figure 2F, G). The stomatal complex type is cyclocytic, with about six subsidiary cells surrounding the guard cells, but the subsidiary cell boundaries are not well differentiated. Abaxial trichome bases are present on the areole‐forming veinlets, seen as darkened circular and small holes, either individually or in clusters of variable number (Figure 4).

Figure 4Light microscopy images of extant *Dryobalanops* species. Three figures are provided for each species to show the abaxial cuticles, closeup views of a giant stoma (arrows), and the adaxial cuticles. (A–C) *Dryobalanops aromatica*, Bernard Lee S.46493, 1983, Sarawak, L.2430341. (D–F) *Dryobalanops beccarii*, Ambriansyah & Z. Arifin AA 887, 1993, Indonesia, L.2430619. (G–I) *Dryobalanops fusca*, P.S. Ashton S.186299, 1963, Sarawak, L.2430559. (J–L) *Dryobalanops keithii*, K. Ogata 10805, 1968, Sabah, L.2430537. (M–O) *Dryobalanops lanceolata*, A. Kadir A 2836, 1950, Sabah, L.2430484. (P–R) *Dryobalanops oblongifolia*, Paul Chai S.19652, 1964, Sarawak, L.2430729. (S–U) *Dryobalanops rappa*, P.S. Ashton BRUN 5105, 1959, Brunei, L.2430702.

**Figure:**
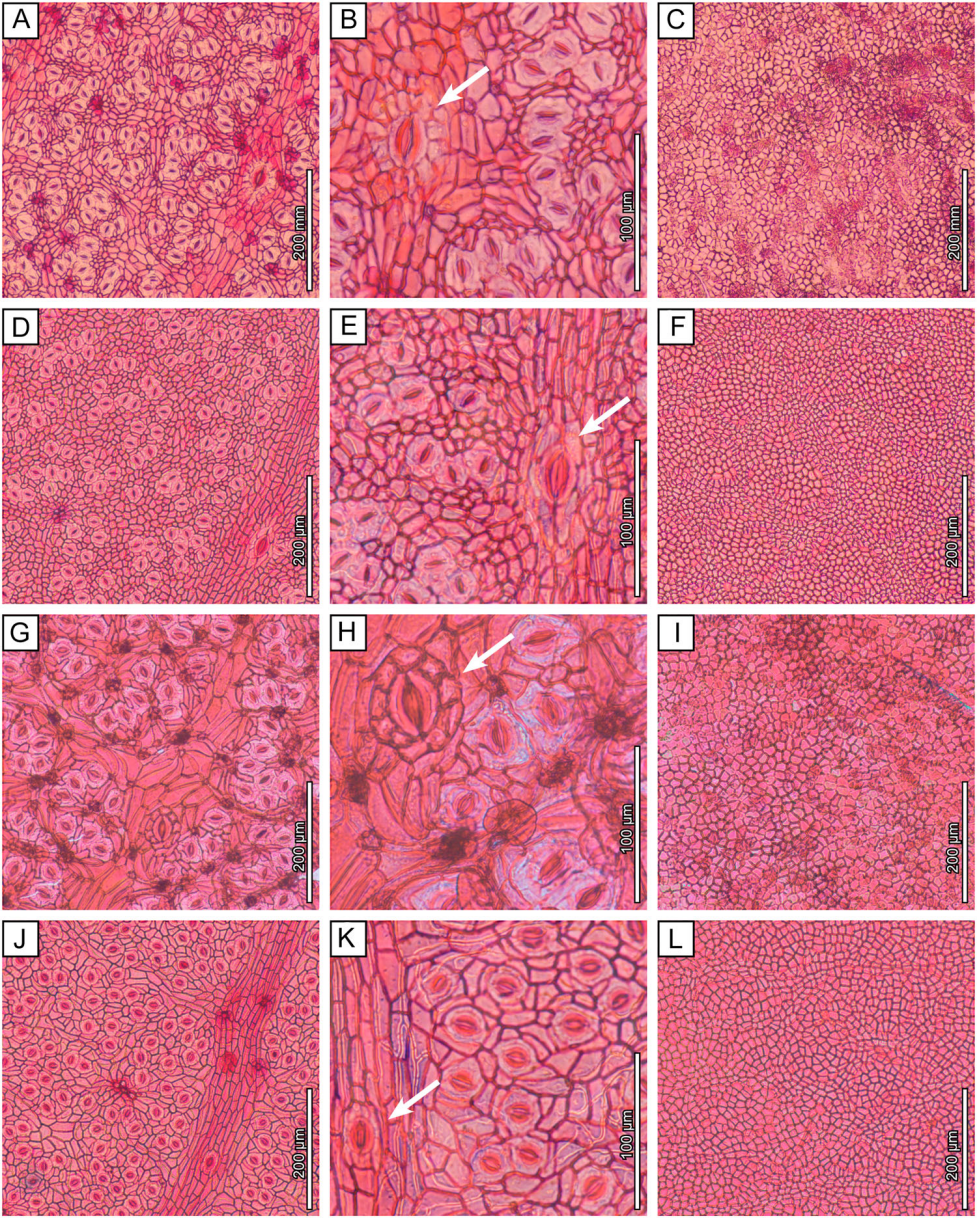

**Figure:**
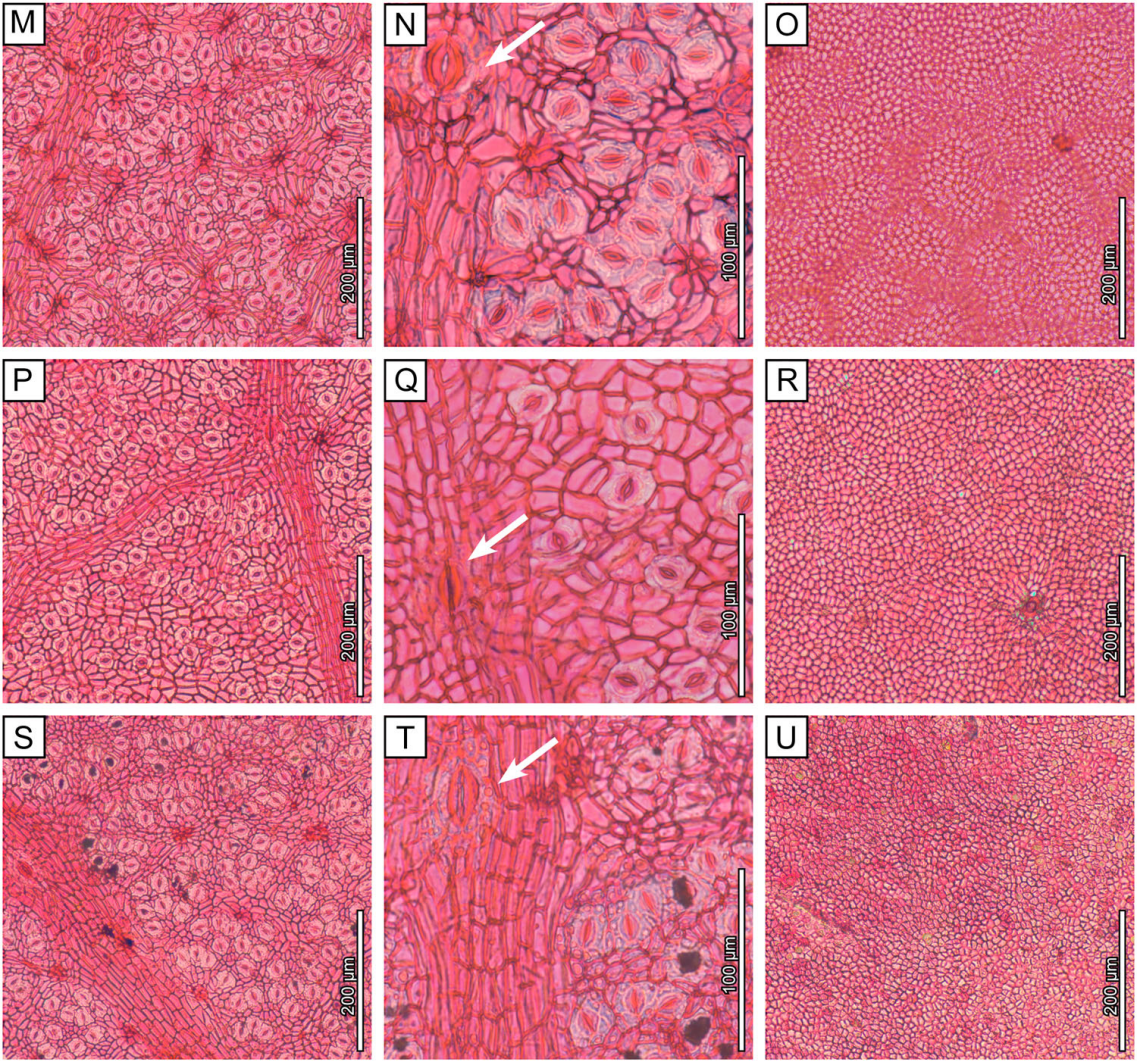

Quantitative characters are similar, overall, among *Dryobalanops* species, with a few exceptions (Table 1). For example, *D. fusca* has apparently larger stomata and lower stomatal density than the other species (Figure 4G, H; Appendix S3A). Abaxial trichome bases are present in all species at varying densities, which suggests that several species previously considered glabrous may in fact have caducous or inconspicuous hairs. Nevertheless, *D. beccarii* and *D. oblongifolia* (Figure 4D–F, P–R) have very few trichome bases on both leaf surfaces and can be considered glabrous.

We also record here, for the first time, that giant stomata are present on the abaxial leaf surfaces of all extant *Dryobalanops* species (Figure 4). They are positioned only on secondary‐intersecondary veins at intervals >6 mm and resemble an enlarged version of normal stomata, clearly having more subsidiary cells (sometimes >10) surrounding the guard cells. These structures most likely share a similar water‐secreting function with hydathodes, which are porous structures that are always open, lack guard cells, and are usually situated at the leaf margins (Bellenot et al., 2022).

## DISCUSSION

### Systematic affinity

Wilf et al. (2022) proposed affinities of the fossils to *Dryobalanops* following detailed comparisons of leaf architecture with several tropical families and genera (e.g., *Syzygium*, *Payena*, *Ouratea*, *Ficus*, *Calophyllum*, Anacardiaceae, Vochysiaceae) that have diverse species with similar leaf shapes and venation. The newly discovered cuticles reveal numerous new characters from microscopic cuticular structures, including the nearly glabrous adaxial surfaces, areolae encircling numerous stomata, cyclocytic stomatal complexes, giant stomata on secondary‐intersecondary veins, and abaxial trichome bases rounded in clusters on the rims of the areolae (Figures 3 and 5; Table 1). This combination of features is found in all seven living species of *Dryobalanops* (Figure 4) and is not known in any of the other genera and families noted to have similar leaves (Wilf et al., 2022). We find the giant stomata placed on the veins to be a particularly distinctive shared character of the *Dryobalanops* species (see below). Other dipterocarp genera with documented cuticles lack this feature, such as *Dipterocarpus*, *Shorea*, and *Hopea* (Khan et al., 2015; Chen et al., 2021), and all other dipterocarp genera lack the fine‐parallel secondary‐intersecondary venation of the fossils. The combined leaf architecture and cuticular features, indistinguishable from living *Dryobalanops* and not found outside Dipterocarpaceae, validate the fossils' generic affinity and allow us to examine their species relationships more closely.

Although leaf venation is similar among all seven living *Dryobalanops* species (their relevant differences were previously discussed by Wilf et al., 2022), four of these species have markedly different shapes and sizes from our fossils (Table 1; Appendix S5): *D. lanceolata* and *D. oblongifolia* have lanceolate and oblong leaves, respectively, as named; *D. aromatica* has orbicular leaves; and *D. keithii* has much larger leaves with lengths >10 cm. A fifth species, *D. beccarii*, is more similar to the fossils but can be excluded due to its blades having very few trichome bases (Figure 4D–F; Table 1) and visually distinct intramarginal veins (Ashton, 2004), whereas our fossils show abundant trichome bases and intramarginal veins placed very close to the margin.

The remaining two species, *D. fusca* (Figure 2D, H; emphasized by Wilf et al. 2022; Critically Endangered) and *D. rappa* (Figure 2C, F, G; Endangered; IUCN, 2024), both Borneo endemics (Ashton, 2004), are each very similar to our fossils because they are tomentose and have overlapping leaf architectural features. *Dryobalanops fusca* is characterized by its tomentum being persistent, even, and dark golden‐brown in color, whereas that of *D. rappa* is at least partially caducous, flocculent, and rufous (Appendix S3; Ashton, 2004). Both species show small polygonal tertiary‐vein fields packed in several rows between secondary‐intersecondary veins, like the fossils (Figure 2). However, the secondary veins of the fossils are more similar to *D. rappa*, which has noticeably stronger intersecondary veins than *D. fusca* (Figure 2). Moreover, the stomata of *D. fusca* are ~70% larger in size and ~50% lower in density than any other living species in the genus (Figure 4; Table 1; Appendix S3), whereas our fossils show comparable stomatal size and density to *D. rappa* (Table 1). The fossils have a trichome base density more similar to *D. fusca* (Table 1), but we consider this a non‐diagnostic character because it is easily influenced by the microenvironment and growth form (Ichie et al., 2016). In sum, our fossils' cuticular features and leaf architecture are both indistinguishable from *D. rappa* (Figures 2, 3, 4, 5; Table 1). Given the geologically young age and the fossil occurrence within the extant range of *D. rappa* (Figure 1), assignment to the living species is justifiable.

### Taxonomic significance of giant stomata

Giant stomata, also referred to as water‐stomata, have been reported in several unrelated plant species in various families of tropical to temperate affinities (Rollet et al., 1990; Boldt and Rank, 2010), including some combretaceous mangroves (Stace, 1966), *Limonia acidissima* L. (Rutaceae; Sitholey and Pandey, 1971), the aquatic fern *Regnellidium diphyllum* Lind. (Marsileaceae; Rao, 1973), *Citrus unshinu* Marc. (Rutaceae; Shiraishi et al., 1975), *Populus nigra* L. (Salicaceae; Russo et al., 2015), *Austrobaileya scandens* C.T.White (Austrobaileyaceae; Rudall and Knowles, 2013), and species of Myrtaceae (Wyk et al., 1982; Bandulska, 2008), Euphorbiaceae (Raju and Rao, 1977), *Nothofagus* (Nothofagaceae; Jordan and Hill, 1999), *Ilex* (Aquifoliaceae; Li et al., 2010), *Zelkova* (Ulmaceae; Denk and Grimm, 2005), *Mangifera* (Anacardiaceae; Sitholey and Pandey, 1971), and *Buxus* (Buxaceae; Huang et al., 2018). However, only those on *Dryobalanops* (Figure 4), *Nothofagus*, and *Mangifera* occur exclusively on veins (Sitholey and Pandey, 1971; Jordan and Hill, 1999); otherwise, they are scattered among the normal stomata accompanying radiating striations on the cuticles (Rudall and Knowles, 2013). In *Nothofagus* and *Buxus*, not every species in the genus possesses giant stomata, suggesting that their presence may have limited taxonomic significance for those genera (Jordan and Hill, 1999; Huang et al., 2018). However, our results demonstrate that giant stomata are a shared character among all *Dryobalanops* species (Figure 4). This contrasts with other Dipterocarpaceae genera, such as *Dipterocarpus*, *Hopea*, and *Shorea*, whose documented cuticles lack giant stomata (Khan et al., 2015; Chen et al., 2021), and no giant stomata have been reported in the remaining genera (e.g., Wilkinson, 1979). Therefore, the presence of giant stomata on the secondary‐intersecondary veins can be regarded as a shared and possibly derived character of *Dryobalanops*.

### *Dryobalanops* fossil record

Fossil records associated with *Dryobalanops* are rare. *Dryobalanops*‐type pollen was reported from the early Eocene of Gujarat, India (Bansal et al., 2022). Prior to the present study and the preceding report (Wilf et al., 2022), the relevant macrofossil record consisted entirely of *Dryobalanoxylon* wood (Ashton et al., 2021). This genus was established for dipterocarpaceous fossil woods that resemble *Dryobalanops* (Den Berger, 1923), although their exclusive affinity to the living genus remains unproven. Recent studies have compiled species lists for these woods, comprising ~20 species, and revised their anatomical characters (Bande and Prakash, 1986; Mandang and Kagemori, 2003; Biswas et al., 2019; Gentis et al., 2022). Here, we briefly review the past distribution of *Dryobalanoxylon* (Figure 1; Appendix S1).

The earliest, and northernmost, *Dryobalanoxylon* records date to the early Miocene in central Myanmar and the late Miocene in the Bengal Basin, whereas the southernmost Miocene occurrence is from Java. This latitudinal distribution is much broader than the present (Figure 1) and possibly associated with the warmer global climate at that time (Westerhold et al., 2020). A few Miocene reports also include southern India and Borneo (Schweitzer, 1958; Kumarasamy and Elayaraja, 2016). During the Pliocene, *Dryobalanoxylon* was reported in southern Vietnam and Java, which suggests a shrinking northernmost distribution. From the Quaternary, *Dryobalanoxylon* was reported in Sumatra and Ambon (Amboina) Island of Sulawesi. Currently, *Dryobalanops* species are only distributed in Borneo, Sumatra, and the Malay Peninsula (all parts of Sunda; Figure 1). *Dryobalanoxylon* has been reported from Thailand (Srivastava and Kagemori, 2001; Kumarasamy and Elayaraja, 2016; Biswas et al., 2019), but we did not find the original documentation of this occurrence (Schweitzer, 1958). We suspect that the previous authors mistook the actual locality, Bangko in Sumatra, Indonesia, for Bangkok, Thailand. As a result, Thailand probably has no confirmed fossil record of *Dryobalanoxylon*. Overall, the *Dryobalanoxylon* fossil record, if representing *Dryobalanops*, shows a distribution that apparently shrank during Neogene global cooling to its current west Malesian range.

### Paleoconservation significance

**Figure 6:**
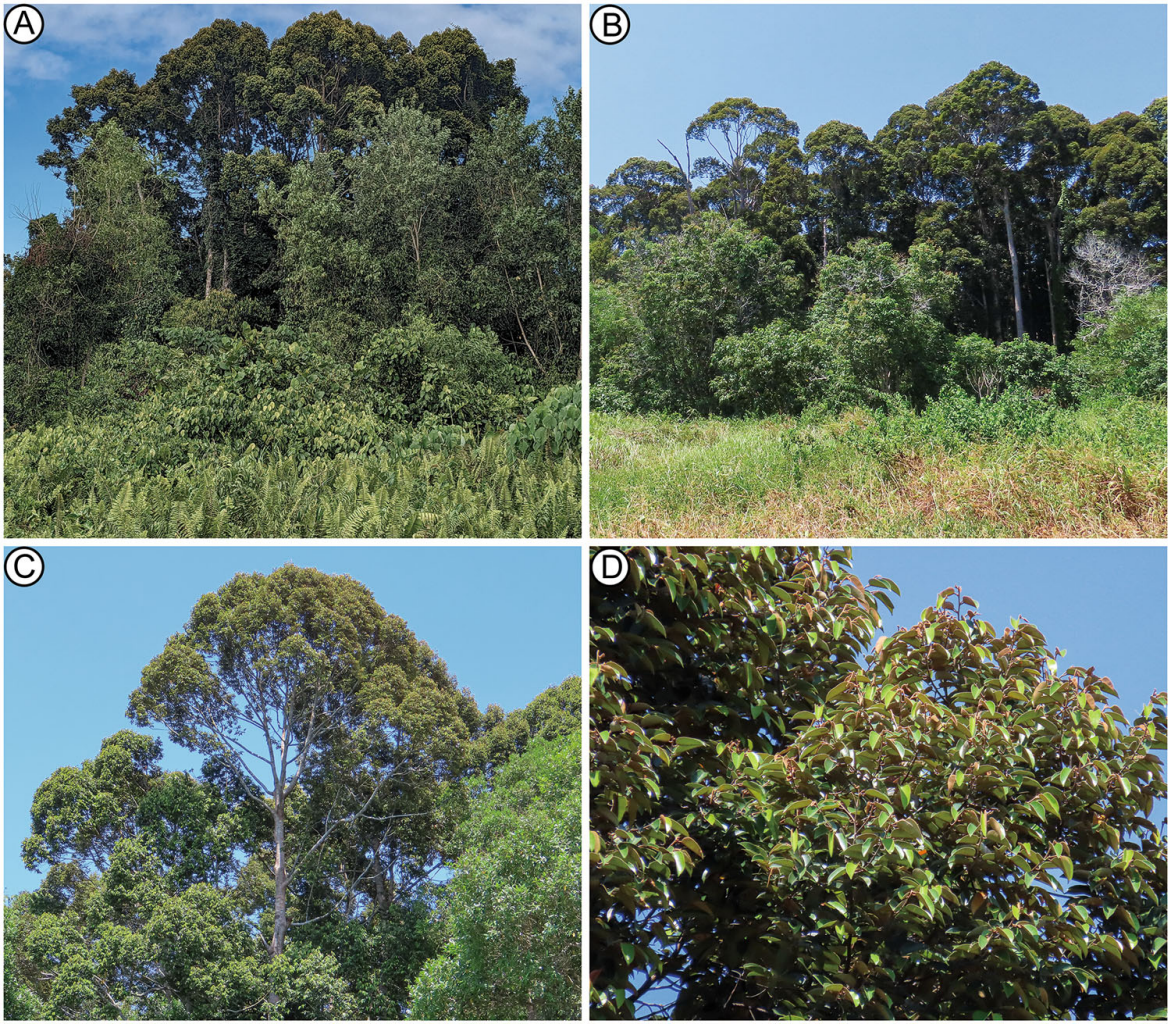
Edge of a fragmentary *Dryobalanops rappa*‐dominant *kerapah* forest in Brunei. (A, B) Canopies of *D. rappa* with disturbed vegetation in the foreground. (C) Emergent canopy of *D. rappa*. (D) Foliage of *D. rappa*, showing natural longitudinal folding of the leaves as seen in the fossils.

*Dryobalanops rappa* (kapur paya) is a large, emergent tree (≤55 m tall) endemic to Brunei and Malaysian Borneo (Figure 1), assessed as Endangered with a decreasing population (Hamidi et al., 2019; IUCN, 2024). The species mostly inhabits wetlands and sandy, acidic soils; it forms dominant stands today in coastal mixed peat swamp and *kerapah* forests of Brunei and Sabah (Figure 6; Ashton, 2004). *Kerapah* (Iban language), the basis of the species epithet *rappa*, refers to a waterlogged habitat having nutrient‐poor and acidic soil at the initial stage of peat formation (Ashton, 2004; Ikbal et al., 2023). The principal threat to the species is massive anthropogenic clearing and conversion of peatlands, and, as a result, their dominance is greatly reduced, especially in Sarawak (Hamidi et al., 2019). The monodominance of the *D. rappa* leaves in the fossil assemblage, associated with *Dipterocarpus* and possible *Shorea*, indicates a dipterocarp‐dominated, most likely peat‐accumulating, coastal rainforest adjacent to the mangrove‐swamp depocenter (Wilf et al., 2022).

Endangered species rarely have a pre‐Holocene fossil record; fossils are known for <9% of threatened mammal species (Plotnick et al., 2016). To our knowledge, endangered plant species having a fossil record include some of the iconic temperate “living fossil” species such as *Metasequoia glyptostroboides* (Yamakawa et al., 2008), *Sequoia sempervirens* (Miki, 1941), and *Ginkgo biloba* (Zhou, 2009) since the Pliocene, but we are not aware of any tropical examples. Thus, our discovery shows new potential for paleobotany to illuminate the evolutionary histories of many other endangered plant species, including the often threatened tropical trees. This knowledge elevates heritage values for conserving ancient lineages, evolutionary processes, and biodiversity that support conservation areas amid the ongoing global biodiversity crisis (Dawson et al., 2011; Barnosky et al., 2017; Fordham et al., 2020).

Our Plio‐Pleistocene fossils demonstrate a previously unknown, prolonged history of the now endangered species *Dryobalanops rappa* dominating coastal, probably peatland rainforests of northern Borneo (Ashton, 2004; Wilf et al., 2022). The Southeast Asian peatlands are vital carbon sinks, accounting for 56% of the world's tropical peatland area and 77% (68.5 Gt) of global peat carbon (Page et al., 2011; Omar et al., 2022). However, anthropogenic disturbances, including agriculture and logging, have left only 36% of Borneo's peat swamp forests remaining and 9% protected (Koh et al., 2011; Posa et al., 2011; Cole et al., 2019; Girkin et al., 2022). Intense fires associated with peatland clearing emit significantly more CO_2_ than typical forest fires (Cochrane et al., 2009; Hooijer et al., 2010). Lowland dipterocarp forests in Borneo are threatened with further decline, and coastal mangrove and peat swamp forests are the most vulnerable habitats (Cannon et al., 2009). Our results show that fossils can be used to unveil the evolutionary history of extant, threatened species and communities, bringing previously unknown heritage values to light for the Asian wet tropics and other endangered ecosystems.

## CONCLUSIONS

We report the first fossils assigned to a living endangered tropical tree species, the dipterocarp *Dryobalanops rappa*, from the Plio‐Pleistocene of Brunei. The fossils were identified through comprehensive comparisons of leaf cuticles and leaf architecture from all extant congeneric species. The diagnostic features of *Dryobalanops* leaf cuticles include nearly glabrous adaxial surfaces, areolae encircling numerous stomata, cyclocytic stomatal complexes, giant stomata on secondary‐intersecondary veins (possibly unique in the family), and abaxial trichome bases rounded and in clusters on the areolar rims. Fossil wood records document an apparent range contraction of probable *Dryobalanops* relatives from a broader Asian distribution to west Malesia from the Miocene to the present. The *D. rappa* fossils from Brunei highlight the evolutionary heritage at risk in Southeast Asia's vanishing rainforests.

## AUTHOR CONTRIBUTIONS

T.W., P.W.: conceptualization, experiments, data analysis, principal draft writing, figure formatting. T.W., M.P.D., P.W., X.Z.: fossil management and imaging. All authors: investigation, resources, validation, text contributions and editing.

## CONFLICT OF INTEREST STATEMENT

Peter Wilf is an Associate Editor of the *American Journal of Botany* but took no part in the peer‐review and decision‐making processes for this paper.

## Supporting information


**Appendix S1.** Macrofossil records of *Dryobalanops* and the wood genus *Dryobalanoxylon*.

**Appendix S2.** Schematic drawing of stomatal complex of *Dryobalanops* for measurement.

**Appendix S3.** Unprepared abaxial leaf surfaces of *Dryobalanops fusca* and *D. rappa* under epifluorescence.

**Appendix S4.** Cuticles of selected unidentified leaves from the Kampong Lugu fossil site.

**Appendix S5.** Leaves of extant *Dryobalanops*.

## Data Availability

Following fieldwork in May–June 2015, the fossil specimens were loaned to the PSU Paleobotany Laboratory (see Wilf et al., 2022). All macrofossil specimens and pollen preparations reported here and in Wilf et al. (2022) were recently returned to UBDH, and the remaining cuticle slides shown here will follow. Full‐resolution image files of the entire macrofossil collection, including all *Dryobalanops* specimens, remain available on Figshare at https://doi.org/10.25452/figshare.plus.16510584 (Wilf et al., 2022).
