# Supplementary material for: Fossils of an endangered, endemic, giant dipterocarp species open a historical portal into Borneo's vanishing rainforests
Source: Am J Bot. 2025 May 8;112(5):e70036. doi: 10.1002/ajb2.70036 (PMC12094065; doi:10.1002/ajb2.70036)
Supplement: Supplementary file 1 — Appendix S1. Macrofossil records of Dryobalanops and the wood genus Dryobalanoxylon. [file AJB2-112-e70036-s002.docx]

APPENDIX S1

Macrofossil records of *Dryobalanops* and the wood genus *Dryobalanoxylon.* Numbers correspond to Figure 1 of the main text. Fossil names follow the International Fossil Plant Names Index (IFPNI, http://ifpni.org). The coordinates were extracted from the cited literature or estimated based on the provided locality information.

| No. | Age | Species | Organ | Location | Reference | Latitude, longitude |
| --- | --- | --- | --- | --- | --- | --- |
| 1 | Pliocene-Pleistocene | *Dryobalanops rappa* | Leaf | Brunei | **This study**; Wilf et al. (2022) | 4.876, 114.802 |
| 2 | Quaternary | *Dryobalanoxylon mirabile* | Wood | Central Sumatra, Indonesia | Schweitzer (1958) | -0.270, 102.237 |
| 3 | Quaternary | *Drobalanoxylon rotundatum* | Wood | Central Sumatra, Indonesia | Schweitzer (1958) | -0.270, 102.237 |
| 4 | Quaternary | *Drobalanoxylon bangkoense* | Wood | Central Sumatra, Indonesia | Schweitzer (1958) | -0.270, 102.237 |
| 5 | Quaternary | *Dryobalanoxylon neglectum* | Wood | Central Sumatra, Indonesia | Schweitzer (1958) | -0.270, 102.237 |
| 6 | Quaternary | *Dryobalanoxylon* cf. *bangkoense* | Wood | Ambon (Amboina) Island, Indonesia | Schweitzer (1958) | -3.698, 128.103 |
| 7 | Late Pliocene | *Dryobalanoxylon sumatrense* | Wood | Central Sumatra, Indonesia | Schweitzer (1958) | -0.270, 102.237 |
| 8 | Pliocene | *Dryobalanoxylon spectabile* | Wood | West Java, Indonesia | Schweitzer (1958) | -6.318, 106.501 |
| 9 | Pliocene | *Dryobalanoxylon tobleri* | Wood | West Java, Indonesia | Schweitzer (1958) | -6.318, 106.501 |
| 10 | Pliocene | *Dryobalanoxylon javanense* | Wood | West Java, Indonesia | Schweitzer (1958) | -6.318, 106.501 |
| 11 | Pliocene | *Dryobalanoxylon lunaris/lunare* | Wood | West Java, Indonesia | Mandang and Kagemori (2003) | -6.383, 106.305 |
| 12 | Pliocene | *Dryobalanoxylon bogorense* | Wood | West Java, Indonesia | Srivastava and Kagemori (2001) | -6.571, 106.633 |
| 13 | Pliocene | *Dryobalanoxylon tambouense* | Wood | Di Linh, Vietnam | Vozenin-Serra (1981) | 11.499, 108.002 |
| 14 | Late Miocene to early Pliocene | *Dryobalanoxylon musperi* | Wood | West Java, Indonesia | Schweitzer (1958) | -6.272, 107.575 |
| 15 | Miocene–Pliocene | *Dryobalanoxylon holdeni/holdeniae* | Wood | Tamil Nadu, India | Awasthi (1969) | 11.957, 79.772 |
| 16 | Miocene–Pliocene | *Dryobalanoxylon indicum* | Wood | Tamil Nadu, India | Awasthi (1969) | 11.957, 79.772 |
| 17 | Late Miocene | *Dryobalanoxylon neosumatrense* | Wood | West Bengal, India | Biswas et al. (2019) | 23.950, 87.667 |
| 18 | Miocene | *Dryobalanoxylon borneense* | Wood | East Kalimantan, Indonesia | Schweitzer (1958) | -0.585, 117.163 |
| 19 | Miocene | *Dryobalanoxylon neyveliensis* | Wood | Tamil Nadu, India | Kumarasamy and Elayaraja (2016) | 11.542, 79.476 |
| 20 | Miocene | *Dryobalanoxylon keralaensis* | Wood | Kerala, India | Awasthi and Ahuja (1982) | 8.750, 76.730 |
| 21 | Miocene | *Dryobalanoxylon tobleri* | Wood | Myanmar | Gottwald (1994) | 22.750, 94.750 |
| 22 | Early Miocene | *Dryobalanoxylon* cf*. javanense*  *Dryobalanoxylon* sp*.* | Wood | Myanmar | Gentis et al. (2022) | 23.273, 94.307 |
| 23 | “Tertiary” | *Dryobalanoxylon tobleri* | Wood | South Sumatra, Indonesia | Den Berger (1923) | -0.270, 102.237 |
| 24 | “Tertiary” or Quaternary | *Dryobalanoxylon khmerinum* | Wood | Cambodia | Boureau (1952); Schweitzer (1958) | 12.463, 106.161 |

**References**

Awasthi, N. 1969. Revision of some dipterocarpaceous woods previously described from the Tertiary of south India. *Journal of Palaeosciences* 18: 226–233.

Awasthi, N., and M. Ahuja. 1982. Investigations of some carbonised woods from the Neogene of Varkala in Kerala Coast. *Geophytology* 12: 245–259.

Biswas, A., M. A. Khan, and S. Bera. 2019. Occurrence of *Dryobalanops* Gaertn. (Dipterocarpaceae) in the late Miocene of Bengal Basin, India and biogeography of the genus during the Cenozoic of Southeast Asia. *Botany Letters* 166: 434–443.

Boureau, E. 1952. Contribution à l’étude paléoxylologiquede l’Indochine. V – Bois fossiles de l’Annam et du Cambodge. *Mémoires du Muséum national d’Histoire naturelle, Série C, Sciencesde la Terre* 2: 1–26.

Den Berger, L. G. 1923. Fossiele houtsoorten uit het Tertiair van Zuid-Sumatra. *Verhandelingen van het Koninklijk Nederlands Geologisch Mijnbouwkundig Genootschap* Geologische Serie 7: 143–148.

Gentis, N., A. Licht, A. Boura, D. Franceschi, Z. Win, D. Aung, and G. Dupont-Nivet. 2022. Fossil wood from the lower Miocene of Myanmar (Natma Formation): palaeoenvironmental and biogeographic implications. *Geodiversitas* 44: 853–909.

Gottwald, H. P. 1994. Tertiäre Kieselhölzer aus dem Chindwinn-Bassin im nordwestlichen Myanmar (Birma). *Documenta naturae*: 1–90.

Kumarasamy, D., and M. Elayaraja. 2016. A new species of *Dryobalanoxylon* Den Berger from the Neyveli Lignite Formation, Tamil Nadu, India. *Journal of Environment and Earth Science* 6: 83–87.

Mandang, Y. I., and N. Kagemori. 2003. A fossil wood of Dipterocarpaceae from Pliocene deposit in the west region of Java Island, Indonesia. *Biodiversitas* 5: 259–275.

Schweitzer, H. J. 1958. Die Fossilen Dipterocarpaceen-Hölzer. *Palaeontographica Abteilung B* 105: 1–66.

Srivastava, R., and N. Kagemori. 2001. Fossil wood of *Dryobalanops* from Pliocene deposits of Indonesia. *Palaeobotanist* 50: 395–401.

Vozenin-Serra, C. 1981. Les structures ligneuses Néogènes du plateau de Di Linh (Sud-Viêtnam). *Palaeontographica Abteilung B*: 136–161.

Wilf, P., X. Zou, M. P. Donovan, L. Kocsis, A. Briguglio, D. Shaw, J. F. Slik, and J. J. Lambiase. 2022. First fossil-leaf floras from Brunei Darussalam show dipterocarp dominance in Borneo by the Pliocene. *PeerJ* 10: e12949.
