# Supplementary material for: Fossils of an endangered, endemic, giant dipterocarp species open a historical portal into Borneo's vanishing rainforests
Source: Am J Bot. 2025 May 8;112(5):e70036. doi: 10.1002/ajb2.70036 (PMC12094065; doi:10.1002/ajb2.70036)
Supplement: Supplementary file 2 — Appendix S2. Schematic drawing of stomatal complex of Dryobalanops for measurement. [file AJB2-112-e70036-s005.docx]

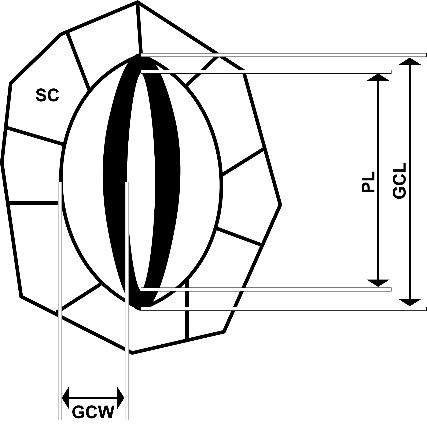


APPENDIX S2

Schematic drawing of stomatal complex of *Dryobalanops* for measurement*.* The measurements were made using light microscope images. PL, pore length; GCL, guard cell length; GCW, guard cell width; sc, subsidiary cell.
