# Supplementary material for: Fossils of an endangered, endemic, giant dipterocarp species open a historical portal into Borneo's vanishing rainforests
Source: Am J Bot. 2025 May 8;112(5):e70036. doi: 10.1002/ajb2.70036 (PMC12094065; doi:10.1002/ajb2.70036)
Supplement: Supplementary file 3 — Appendix S3. Unprepared abaxial leaf surfaces of Dryobalanops fusca and D. rappa under epifluorescence. [file AJB2-112-e70036-s001.docx]

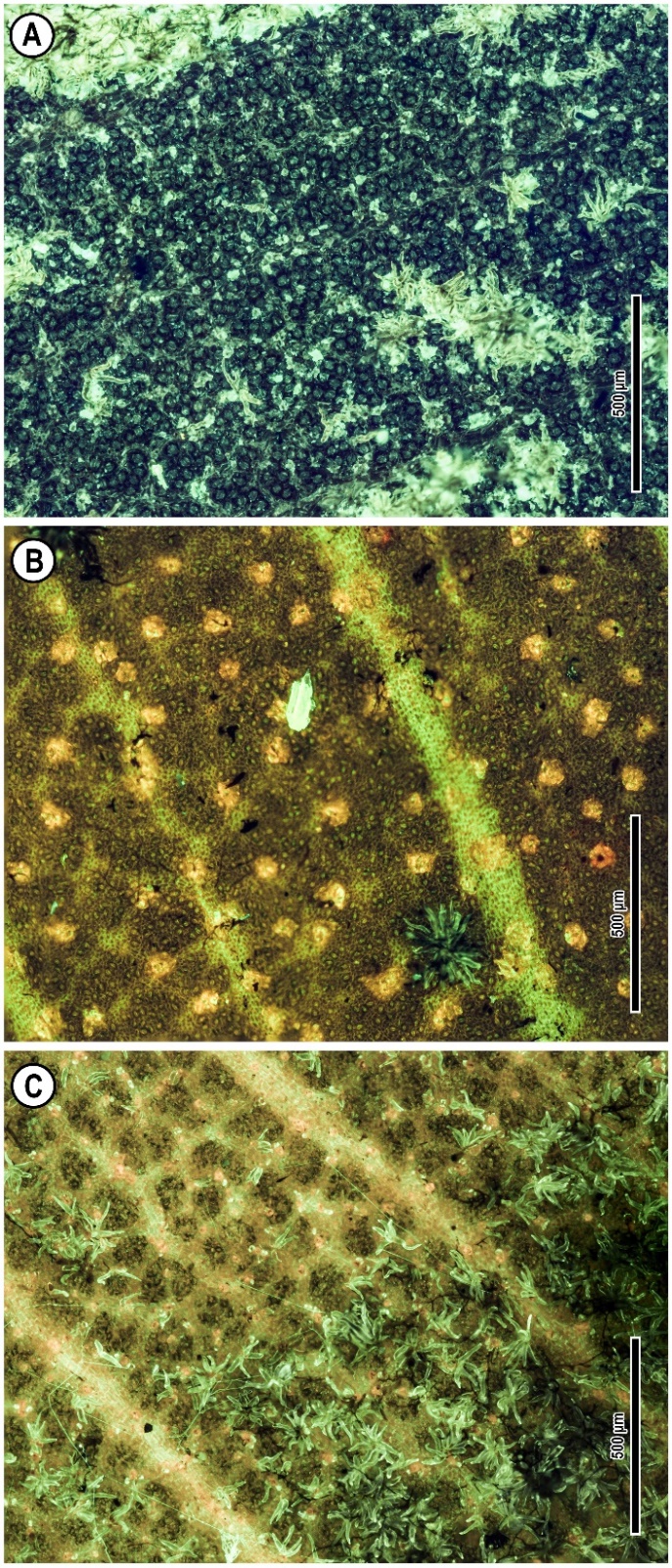


APPENDIX S3

Unprepared abaxial leaf surfaces of *Dryobalanops fusca* and *D. rappa* under epifluorescence*.* **(**A) *D. fusca* (Voucher: Ariffin 9625, 1959, Sarawak, A02566692), showing an exposed area in the midst of dense hair tufts and relatively larger stomata. (B) *D. rappa* (Voucher: S. Tong S34166, 1974, Sarawak, A02566817), showing an area without the prevalence of hair tufts (caducous). (C) *D. rappa* (Voucher: K.M. Wong WKM917, 1989, Brunei, A02566800), showing an area with hair tufts, less dense than *D. fusca*.
