# Supplementary material for: Fossils of an endangered, endemic, giant dipterocarp species open a historical portal into Borneo's vanishing rainforests
Source: Am J Bot. 2025 May 8;112(5):e70036. doi: 10.1002/ajb2.70036 (PMC12094065; doi:10.1002/ajb2.70036)
Supplement: Supplementary file 4 — Appendix S4. Cuticles of selected unidentified leaves from the Kampong Lugu fossil site. [file AJB2-112-e70036-s003.docx]

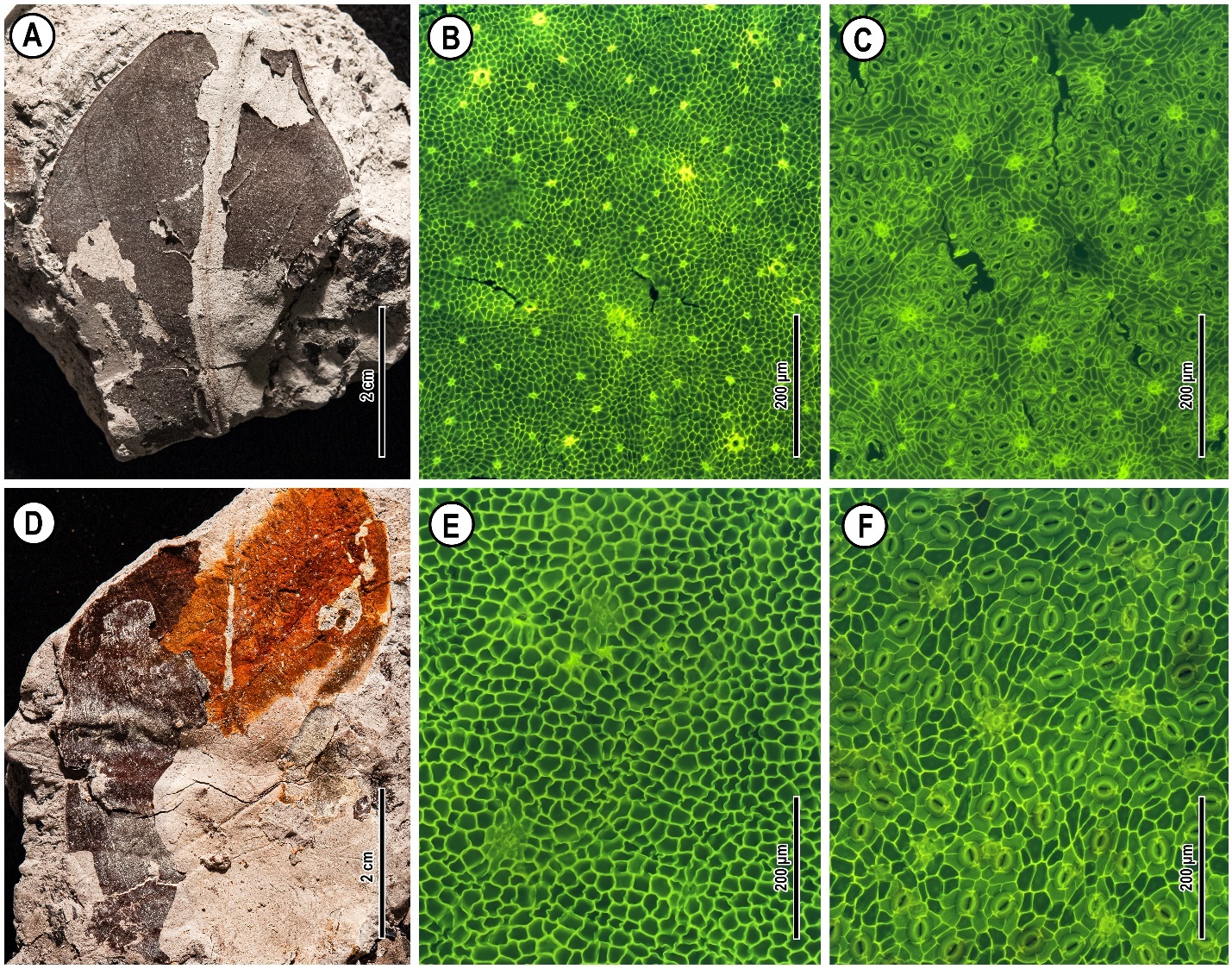


APPENDIX S4

Cuticles of selected unidentified leaves from the Kampong Lugu fossil site, demonstrating the potential for future cuticular studies on this flora. (A–C) UBDH F00218. (D–F) UBDH F00176. (B, E) Adaxial. (C, F) Abaxial.
