# Supplementary material for: Fossils of an endangered, endemic, giant dipterocarp species open a historical portal into Borneo's vanishing rainforests
Source: Am J Bot. 2025 May 8;112(5):e70036. doi: 10.1002/ajb2.70036 (PMC12094065; doi:10.1002/ajb2.70036)
Supplement: Supplementary file 5 — Appendix S5. Leaves of extant Dryobalanops. [file AJB2-112-e70036-s004.docx]

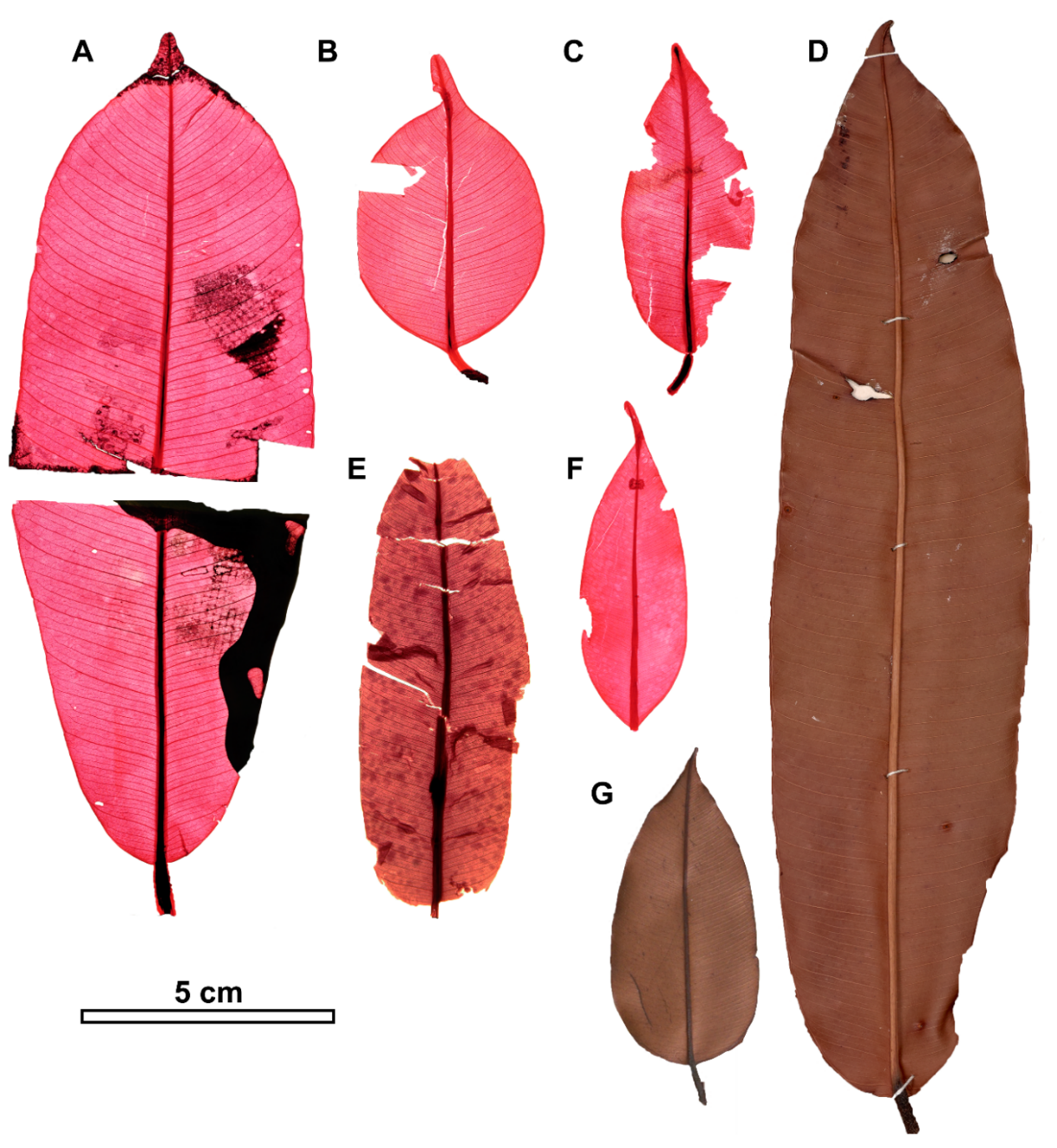


APPENDIX S5

Leaves of extant *Dryobalanops.* (A) *D. keithii* (National Cleared Leaf Collection, Wolfe 7924a, b. Source voucher: G.H.S. Wood, San 3966, 1954, Sabah, A02566699). (B) *D. aromatica* (National Cleared Leaf Collection, Wolfe 7921. Source voucher: I. Ahmad, Kepong 75015, 1955, Malay Peninsula, A02566652). (C) *D. fusca* (National Cleared Leaf Collection, Wolfe 7923. Source voucher: Ariffin 9625, 1959, Sarawak, A02566692). (D) *D. oblongifolia* (Voucher: F. Chu, Adenan, & Sibat S.23176, 1967, Sarawak, A02566775). (E) *D. lanceolata* (National Cleared Leaf Collection, Hickey 1560. Source voucher: Kadir, A22, 1947, Sabah, US2317065). (F) *D. beccarii* (National Cleared Leaf Collection, Wolfe 7922. Source voucher: Esah, SAN 35707, 1963, Sabah, A02566689). (G) *D. rappa* (Voucher: Boschproefstation BB.7680, 1924, West Borneo, L.2430735).

Image information: Cleared leaf specimens (A–C, E, F) from the National Cleared Leaf Collection are housed in the Division of Paleobotany, National Museum of Natural History, Smithsonian Institution, and the images are available in an open-access dataset (Wilf et al., 2021). D, downloaded from <https://kiki.huh.harvard.edu/databases/specimen_search.php?barcode=A02566775> and reproduced with permission. G, downloaded from <https://bioportal.naturalis.nl/nl/specimen/L.2430735> under Creative Commons License CC0 1.0 license.

Wilf, P., Wing, S.L., Meyer, H.W., Rose, J.A., Saha, R., Serre, T., Cúneo, N.R., Donovan, M.P., Erwin, D.M., Gandolfo, M.A., Gonzalez-Akre, E., Herrera, F., Hu, S., Iglesias, A., Johnson, K.R., Karim, T.S., Zou, X., 2021. An image dataset of cleared, x-rayed, and fossil leaves vetted to plant family for human and machine learning. *PhytoKeys* 187, 93–128.
